# Melanopsin as a Sleep Modulator: Circadian Gating of the Direct Effects of Light on Sleep and Altered Sleep Homeostasis in *Opn4^−/−^* Mice

**DOI:** 10.1371/journal.pbio.1000125

**Published:** 2009-06-09

**Authors:** Jessica W. Tsai, Jens Hannibal, Grace Hagiwara, Damien Colas, Elisabeth Ruppert, Norman F. Ruby, H. Craig Heller, Paul Franken, Patrice Bourgin

**Affiliations:** 1 Department of Biology, Stanford University, Stanford, California, United States of America; 2 Department of Clinical Biochemistry, Rigshopitalet, Copenhagen, Denmark; 3 Laboratory of Rhythms - CNRS UMR 7168/LC2, Louis Pasteur University and Department of Neurology - School of Medicine, Strasbourg, France; 4 Center for Integrative Genomics, University of Lausanne, 1015 Lausanne-Dorigny, Switzerland; Oxford University, United Kingdom

## Abstract

Light influences sleep and alertness either indirectly through a well-characterized circadian pathway or directly through yet poorly understood mechanisms. Melanopsin (Opn4) is a retinal photopigment crucial for conveying nonvisual light information to the brain. Through extensive characterization of sleep and the electrocorticogram (ECoG) in melanopsin-deficient (*Opn4^−/−^*) mice under various light–dark (LD) schedules, we assessed the role of melanopsin in mediating the effects of light on sleep and ECoG activity. In control mice, a light pulse given during the habitual dark period readily induced sleep, whereas a dark pulse given during the habitual light period induced waking with pronounced theta (7–10 Hz) and gamma (40–70 Hz) activity, the ECoG correlates of alertness. In contrast, light failed to induce sleep in *Opn4^−/−^* mice, and the dark-pulse-induced increase in theta and gamma activity was delayed. A 24-h recording under a LD 1-h∶1-h schedule revealed that the failure to respond to light in *Opn4^−/−^* mice was restricted to the subjective dark period. Light induced c-Fos immunoreactivity in the suprachiasmatic nuclei (SCN) and in sleep-active ventrolateral preoptic (VLPO) neurons was importantly reduced in *Opn4^−/−^* mice, implicating both sleep-regulatory structures in the melanopsin-mediated effects of light. In addition to these acute light effects, *Opn4^−/−^* mice slept 1 h less during the 12-h light period of a LD 12∶12 schedule owing to a lengthening of waking bouts. Despite this reduction in sleep time, ECoG delta power, a marker of sleep need, was decreased in *Opn4^−/−^* mice for most of the (subjective) dark period. Delta power reached after a 6-h sleep deprivation was similarly reduced in *Opn4^−/−^* mice. In mice, melanopsin's contribution to the direct effects of light on sleep is limited to the dark or active period, suggesting that at this circadian phase, melanopsin compensates for circadian variations in the photo sensitivity of other light-encoding pathways such as rod and cones. Our study, furthermore, demonstrates that lack of melanopsin alters sleep homeostasis. These findings call for a reevaluation of the role of light on mammalian physiology and behavior.

## Abstract 2

Analyses in mice deficient for the blue-light-sensitive photopigment melanopsin show that direct effects of light on behavior and EEG depend on the time of day. The data further suggest an unexpected role for melanopsin in sleep homeostasis.

## Author Summary

Light affects sleep in two ways: indirectly through the phase adjustment of circadian rhythms and directly through nonvisual mechanisms that are independent of the circadian system. The direct effects of light include the promotion of sleep in night-active animals and of alertness in diurnal species. We analyzed sleep and the electroencephalogram (EEG) under various light–dark regimens in mice lacking melanopsin (*Opn4* ^−/−^), a retinal photopigment crucial for conveying light-level information to the brain, to determine the role of melanopsin, as opposed to rod and cones, in mediating these direct effects of light. We show that melanopsin mediates the direct effects of light during the subjective dark period, whereas rods and cones contribute to these effects in the light period. Our finding that “sleep-active” (i.e., galanin-positive) neurons of the anterior hypothalamus are not activated by light in *Opn4* ^−/−^ mice suggests that these neurons are part of the circuitry whereby light promotes sleep. Also, the alerting effects of transitions into darkness were less pronounced in *Opn4* ^−/−^ mice judged on the reduced increase in EEG theta and gamma activity. Finally, and unexpectedly, the rate at which the need for sleep, quantified as EEG delta power, accumulated during wakefulness was found to be reduced in *Opn4* ^−/−^ mice both during baseline and sleep deprivation conditions, implicating a photopigment in the homeostatic regulation of sleep. We conclude that melanopsin contributes to the direct effects of light and darkness, and in interaction with circadian and homeostatic drive, determines the occurrence and quality of both sleep and waking. If confirmed in humans, our observations will have applications for the clinical use of light as well as for societal lighting conditions.

## Introduction

Light exerts strong effects on human physiology and behavior, including entrainment of circadian rhythms [1]–[3], suppression of melatonin release [4], regulation of heart rate and body temperature [5], alertness [6], and cognition [7]. The effects of light on behavior have been classified either as indirect, shifting the phase of the circadian rhythm (photic entrainment), or as direct, affecting behavior in a circadian-independent fashion, such as occurs in light avoidance in nocturnal species (masking). Processing of photic information has been studied extensively in the context of circadian biology with an emphasis on the nonvisual effects of light [8]–[10] mediated by melanopsin, a photopigment involved in irradiance level detection [11]–[13]. In mammals, melanopsin is exclusively expressed in retinal ganglion cells (RGCs) [14] and retinal pigment epithelium [15]. Melanopsin plays a major role in the photic phase shifting of circadian rhythms [16],[17]. The role of light in the circadian regulation of sleep and wakefulness and the pathways by which melanopsin-containing RGCs influence the circadian system, particularly the suprachiasmatic nuclei (SCN), are well documented [18],[19]. In contrast, the noncircadian, direct effects of light on sleep and wakefulness as well as the pathways relaying these effects remain poorly understood. Direct effects of light are difficult to distinguish from visual and circadian influences. Such effects are mainly known to acutely promote alertness in day-active species and sleep in night-active species.

The purpose of this study was to take advantage of mice lacking melanopsin (*Opn4^−/−^*) to provide an in-depth analysis of the direct effects of light on sleep, wakefulness, and the electrocorticogram (ECoG) and how these effects are modulated by time of day under various light–dark (LD) regimes. Two recent studies [20],[21], performed concomitantly to ours, confirmed that in the absence of melanopsin a single light pulse, presented during the dark period, failed to induce sleep. This led to the conclusion that melanopsin mediates the direct effects of light on sleep [20],[21]. By probing the effects of light on sleep across the 24-h day, we demonstrate here that the failure to respond to light in *Opn4^−/−^* mice was restricted to the dark period, implicating other light-encoding pathways in mediating the direct effects of light at other times of day. Another novel aspect of our study is the finding that melanopsin plays a role in shaping rhythmic ECoG activity. In particular, the amplitude of the sleep-wake–dependent changes in ECoG delta power, a marker of sleep need, was reduced in *Opn4^−/−^* mice compared to that of wild-type littermate controls. Finally, to gain insight into the pathways relaying these direct effects, we used c-Fos immunohistochemistry in SCN neurons and in galaninergic “sleep-active neurons” of the ventrolateral preoptic (VLPO) area: key circadian and sleep-promoting structures in the hypothalamus, respectively. The VLPO receives direct projections from melanopsin-containing RGCs [22]–[24] and thus represents a candidate brain area to mediate the direct effects of light on sleep.

## Results

We compared the direct effects of light between mice carrying a targeted disruption of the melanopsin gene (*Opn4^−/−^*; see [17] and Text S1 for details) and their wild-type, littermate controls (*Opn4^+/+^*). We used various lighting schedules to evaluate the effect of light on sleep and the ECoG (for an overview, see Figure S1). Among these schedules were the standard baseline LD 12-h∶12-h (12∶12) schedule the mice were kept under, a single 1-h light and a single 1-h dark pulse given during the habitual 12-h dark and light periods, respectively, and 24 h under a LD 1-h∶1-h (1∶1) cycle.

### Melanopsin Modulates the Direct Effects of Light on Sleep and the ECoG

In a first set of experiments, the acute effects of light and darkness on sleep and the ECoG were investigated by exposing mice of both genotypes to a single, 1-h pulse of light administered 3 h after the habitual LD transitions; i.e., Zeitgeber time (ZT)15–16 (with ZT0 referring to the time of light onset) and to a 1-h dark pulse given 3 h after light onset (i.e., ZT3–4). Mice were otherwise kept under LD 12∶12 conditions, and at least 14 d separated any two recording conditions.

**Figure 1:**
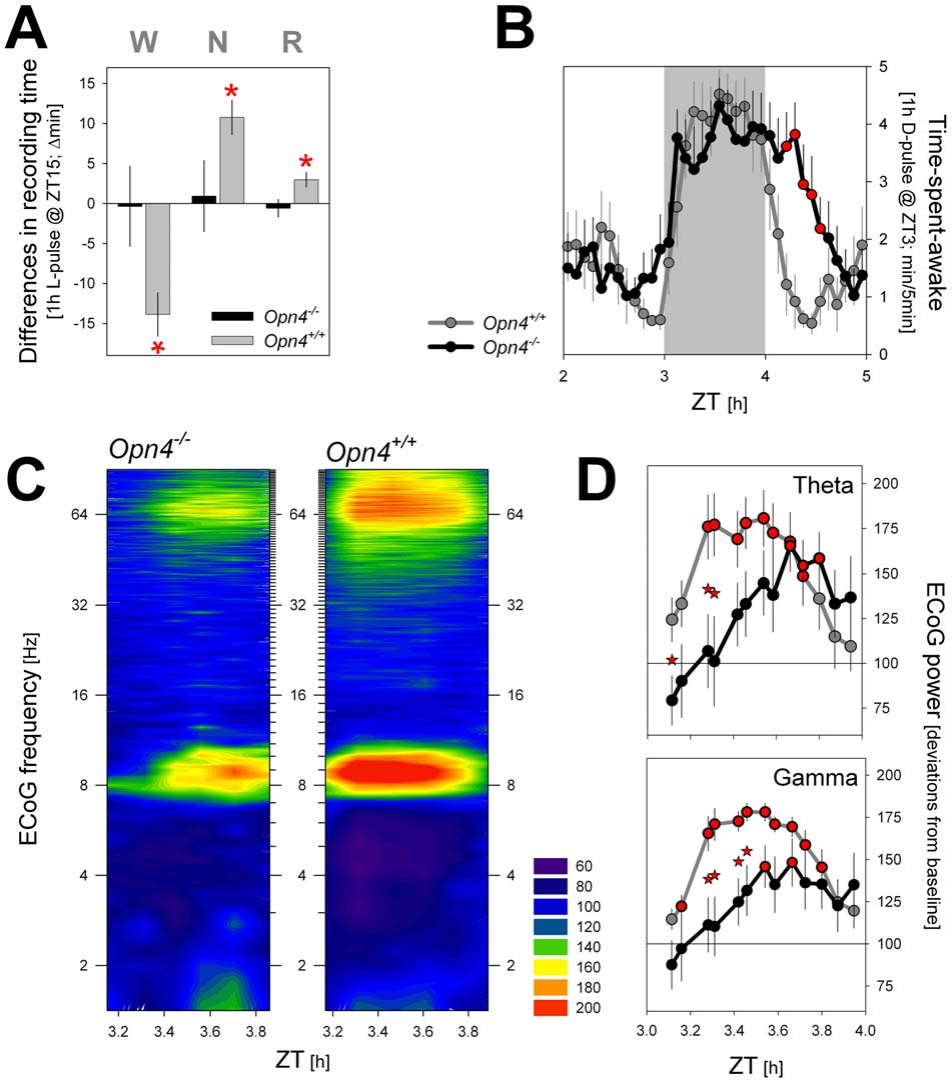
Direct effects of a 1-h light (L) and a 1-h dark (D) pulse on the sleep–wake distribution (A and B) and the waking ECoG (during the 1-h D pulse; panels [C and D]) under an LD 12∶12 schedule. (A) The L pulse, administered during the habitual dark period (ZT 15–16), induced NREM (N) and REM (R) sleep at the expense of wakefulness (W) in *Opn4^+/+^* mice (*p*<0.05; post hoc paired *t*-tests). No response was observed in *Opn4^−/−^* mice. Values during the 1-h L pulse were compared to baseline values obtained in a 3-h window centered around the time of the pulse (ZT 14–17). (B) Time course of time spent awake across the 1-h D pulse, administered during the habitual light period (ZT3–4). Waking values (waking minutes/5-min intervals) over 3 h (1-h light before and 1-h light after D pulse) and centered on the D pulse (ZT3–4; grey area). Note the delayed response in *Opn4^−/−^* mice after the D pulse when light is applied again. Melanopsin-deficient mice stay awake and return to sleep only after 35 min. Red asterisks (*) denote 5-min intervals with significant genotype effects (*p*<0.05; post hoc *t*-tests). (C) Heat map of the time course of spectral changes in the waking ECoG during the 1-h D pulse. ECoG power density was expressed as percentage of individual waking ECoG spectra obtained during the baseline light period ( = 100%). Warmer colors denote relative increases, colder colors, decreases versus baseline ECoG activity. Relative spectral profiles were calculated over 10 min of waking at 5-min increments (i.e., 13 spectra/hour). (D) as in (C) but summarized for the theta (7–10 Hz) and gamma (40–70 Hz) frequency bands. Note the smaller and delayed activation in *Opn4^−/−^* mice. Asterisks indicate significant genotype differences (*p*<0.05; post hoc *t*-tests); red-filled circles indicate significant differences from baseline (*p*<0.05; post hoc paired *t*-tests). Values represent means±SEM (*n* = 6 and 7, for *Opn4^+/+^* and *Opn4^−/−^*, respectively).

In *Opn4^+/+^* mice, the light pulse readily increased the amounts of both rapid eye movement (REM) and non-REM (NREM) sleep at the cost of wakefulness, whereas the same light pulse in *Opn4^−/−^* mice failed to affect time spent in either behavioral state for the duration of the light pulse (Figure 1A). This lack of a response demonstrates that, at least at this time of day (see LD 1∶1 results below), melanopsin contributes significantly to the acute effect of light on sleep.

Light-to-dark transitions induce waking and alertness in nocturnal rodents. Accordingly, in *Opn4^+/+^* mice, a 1-h dark pulse induced an immediate increase in time spent awake (Figure 1B), and the ECoG during wakefulness showed a rapid and prolonged induction in ECoG theta (7–10 Hz) and gamma (40–70 Hz) activity (Figure 1C and 1D), the ECoG correlates of exploratory behavior and alertness in rodents [25],[26]. The dark pulse also induced waking in *Opn4^−/−^* mice. Although their immediate response was somewhat delayed (compare genotypes for the increase in waking in the 5 min before and after dark onset in Figure 1B), the hourly amount of waking (and sleep) did not differ between genotypes (unpublished data). The changes in ECoG theta and gamma activity that follow the transition into darkness seemed, however, to be modulated by melanopsin because they were delayed by ca. 25 min (Figure 1C and 1D). Upon restoring the normal light condition 1 h later, *Opn4^−/−^* mice stayed awake longer, consistent with the light-pulse results (Figure 1B and 1A).

### The Effects of Light and Dark Pulses on Sleep and Waking Vary with Time of Day

**Figure 2:**
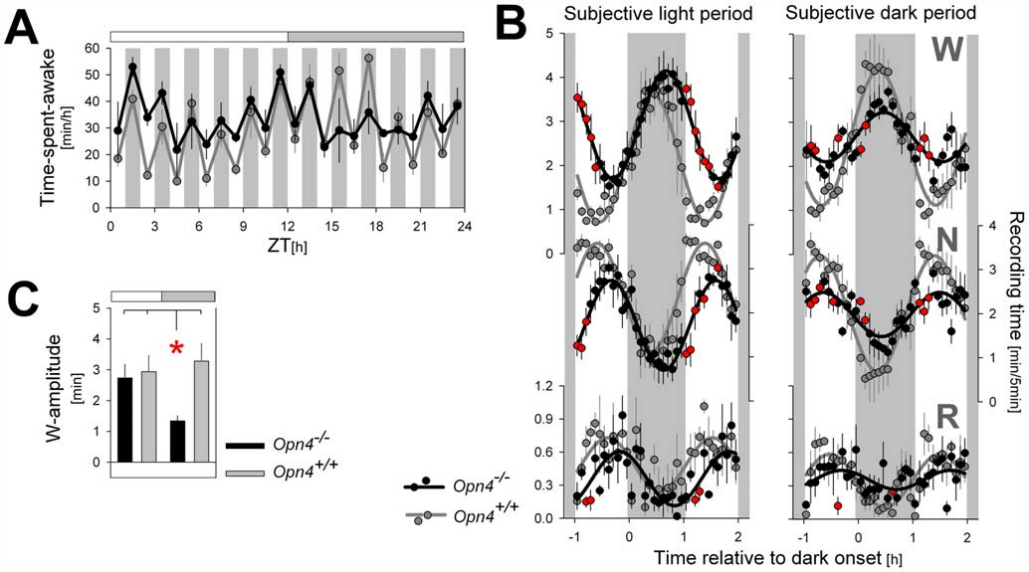
Direct effects of 1-h light (L) and 1-h dark (D) pulses on the sleep–wake distribution under a LD 1∶1 schedule. (A) Hourly mean values of wakefulness during the LD 1∶1 cycle. Hourly L pulses suppressed wakefulness, but this effect seemed to vary with time of day in *Opn4^−/−^* mice and was especially small between ZT15 and ZT21. Grey horizontal bars mark the subjective dark period (i.e., the 12-h dark period of the preceding days under LD 12∶12). (B) Average time course of the LD-induced changes in wakefulness (W; upper), NREM sleep (N; middle), and REM sleep (R; lower panels) during the 12-h subjective light (left) and subjective dark (right) period. Values represent means (±SEM) over 5-min intervals in the hour preceding, during, and following the six 1-h dark pulses in the subjective light and dark periods, respectively. A three-way ANOVA with factors “genotype,” “time of day” (subjective light versus subjective dark period), and “time course” (5-min values) revealed that for W and N, time course was significantly affected by time of day and genotype (interactions: genotype×time of day: W and N: *p*<0.0001; R: *p* = 0.017; genotype×time course: W and N: *p*<0.0001; R: *p* = 0.14; time of day×time course: W: *p* = 0.019; N: *p* = 0.048; R: *p* = 0.18). Red filled circles denote 5-min intervals with significant genotype effects (*p*<0.05; post hoc *t*-tests). Black (*Opn4^−/−^*) and grey (*Opn4^+/+^*) sine waves represent best fits to the data points. (C) Changes in the time course observed in (B) were summarized and quantified by determining the amplitude of sine waves of best fit for each individual mouse (see Text S1 for details). The thus estimated amplitude of the LD-induced changes in wakefulness was affected by time of day (two-way ANOVA interaction genotype×time of day *p* = 0.033) and was significantly smaller in *Opn4^−/−^* mice during the subjective dark period compared to the subjective light period and compared to the values obtained in wild-type mice in both conditions (red asterisk; post hoc *t*-tests; *p*<0.05).

The single pulses of light and darkness probed their effects on sleep and waking at specific times of day only. To obtain a more complete account of the genotype differences of these interventions, we maintained mice under a short, 1-h LD cycle (LD 1∶1) for a 24-h period. This lighting schedule was used first in the rat to investigate the direct effects of light on sleep across the circadian cycle [27]. Also under these conditions, 1-h periods of light induced sleep, and 1-h periods of darkness favored wakefulness. Irrespective of the effect of this ultradian LD cycle on sleep and wakefulness, a circadian modulation of the average levels of sleep and waking reached within the subjective 12-h light period and subjective 12-h dark period is preserved in wild-type mice (Figure 2A; Table 1). The protocol has the potential drawbacks that light given at any one hour might influence its effects during subsequent hours and that animals could entrain their ultradian sleep–wake organization to this schedule. We did not see evidence of the former effect as the relative effect of the LD alteration on time spent awake did not vary with time of day in wild-type mice (Figure 2A–2C), but after the initial three cycles (i.e., after ZT6), wild-type mice seemed capable of anticipating dark onset, because increases in wakefulness started preceding it (Figures 2B and S2).

**Table 1:** Time spent asleep and awake under the LD 12∶12 and LD 1∶1 schedules.

Schedule	Time spent	*Opn4* Genotype	Waking (h)	NREMS (h)	REMS (min)
LD12∶12	12-h light period	^−/−^	4.80±0.21^a^	5.92±0.18^a^	77.1±2.6
		^+/+^	3.74±0.17	6.95±0.17	77.8±11.1
	12-h dark period	^−/−^	6.59±0.20^b^	4.56±0.18^b^	50.8±2.3^b^
		^+/+^	6.79±0.37^b^	4.51±0.33^b^	41.9±6.5^b^
	LD difference	^−/−^	1.80±0.17^a^	1.36±0.14^a^	26.3±3.2
		^+/+^	3.04±0.34	2.44±0.29	35.9±6.6
	24 h	^−/−^	11.39±0.37	10.48±0.33	127.9±3.6
		^+/+^	10.53±0.47	11.47±0.44	119.7±16.9
LD1∶1	*Subjective* 12-h light period	^−/−^	6.96±0.34^a^	4.19±0.29^a^	50.8±3.8^a^
		^+/+^	5.15±0.48	5.66±0.40	71.3±5.1
	*Subjective* 12-h dark period	^−/−^	6.44±0.26	4.71±0.21	51.2±4.7
		^+/+^	6.48±0.18^b^	4.69±0.16	49.9±1.7^b^
	24 h	^−/−^	13.40±0.36^a^	8.9±0.34	101.9±3.4^a^
		^+/+^	11.63±0.65	10.35±0.56	121.2±6.7
	12 1-h light periods	^−/−^	5.51±0.29^a^	5.47±0.23^a^	61.3±5.2^a^
		^+/+^	3.53±0.36	7.13±0.29	80.5±4.2
	12 1-h dark periods	^−/−^	7.89±0.25^b^	3.44±0.23^b^	40.6±3.6^b^
		^+/+^	8.10±0.87^b^	3.22±0.76^b^	40.7±7.1^b^

Under both schedules, *Opn4^−/−^* mice displayed significantly less NREM sleep (NREMS; and were awake more) than *Opn4^+/+^* mice during the (subjective) 12-h light (L) periods. This resulted in a significantly decreased amplitude of the circadian distribution (LD difference) of NREMS time (and waking) in *Opn4^−/−^* mice. Under the LD 1∶1 schedule, *Opn4^−/−^* mice slept less compared to *Opn4^+/+^* mice during all 12 1-h L pulses of the entire 24-h d. Also under the LD1∶1 schedule, the circadian distribution of sleep and waking observed under the LD 12∶12 condition is maintained only in *Opn4^+/+^* mice (subjective 12-h L period, ZT0–12, compared to the. subjective 12-h dark (D) period, ZT12–24).

a Indicates significant genotype differences.

b The difference between (subjective) 12-h D and L periods for each lighting schedule and genotype (*p*<0.05; post hoc *t*-test). Values represent means±SEM (LD 12∶12 *n* = 9/genotype; LD 1∶1 *n* = 3 and 4, for *Opn4^+/+^* and *Opn4^−/−^*, respectively).

Visual inspection of the hourly values of wakefulness reached under the LD 1∶1 protocol suggests that *Opn4^−/−^* mice have a reduced response to light in the (subjective) dark period (Figure 2A); i.e., the time at which we and others [20],[21] administered the single 1-h light pulse (see above). This reduction was, however, not observed at other times of day, and the LD-induced changes in time spent awake did not differ from wild-type mice during the subjective light period (Figure 2A–2C). The capacity of the 1-h LD cycles to alter the sleep–wake distribution was further analyzed by aligning the subsequent 1∶1-h LD cycles (Figures 2B and S2). This time course analysis revealed that, as was observed after the single dark-pulse experiment (Figure 1B), *Opn4^−/−^* mice were again slower in initiating sleep after light onset (Figure 2B). Analyses of variance confirmed that the time course of the LD-induced changes in wakefulness and NREM sleep varied significantly according to genotype and time of day (i.e., 12-h subjective light period versus 12-h subjective dark period; Figure 2B). Changes in the time course observed in Figure 2B were summarized by estimating the amplitude of sine waves of best fit for individual mice (Figure 2C; see Text S1 for details). This analysis also confirmed that genotype affected the light-induced changes in wakefulness only during the subjective dark period (Figure 2C). Especially between ZT15–21, the increase of wakefulness during the 1-h dark intervals compared to the 1-h mean value in the preceding light intervals was no longer significant in *Opn4^−/−^* mice (+4.2±4.1 min; *p* = 0.38 and +26.2±5.3 min; *p* = 0.038; for *Opn4^−/−^* and *Opn4^+/+^* mice, respectively; paired *t*-tests), and this increase differed between genotypes (one-way ANOVA; *p* = 0.020). It thus seems that only during this time melanopsin contributed significantly to the direct effects of light on wake and sleep duration.

### Light-Induced c-Fos Expression in SCN Neurons and Galaninergic Neurons of the VLPO

**Figure 3:**
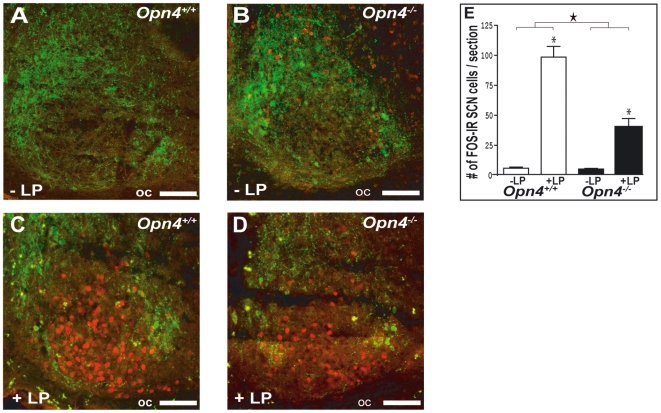
Effects of a 1-h light (L) pulse on c-Fos immunoreactivity in the SCN. (A–D) Effects of the L pulse administered during the habitual dark period (ZT15–16) on c-Fos immunoreactivity in the SCN of *Opn4^+/+^* and *Opn4^−/−^* mice. Few c-Fos immunoreactive cells (labeled red) are found in the dorsomedial (arginine-vasopressin [AVP]-containing) SCN (labeled green) in control (no L pulse) animals (A and B). Light induced c-Fos in the retino-recipient zone of the SCN in *Opn4^+/+^* (C) and to a lesser extend in *Opn4^−/−^* mice (D). oc; optic chiasm. Scale bars in (A–D) indicate 75 µm. (E) Number of c-Fos immunoreactive neurons in the SCN. The L pulse induced c-Fos in the retino-recipient part of the SCN in both genotypes (two-way ANOVA, light pulse effect: *p*<0,001; an asterisk (*) indicates post hoc Fisher PLSD: *p*<0.05), but this light induced c-Fos immunoreactivity is significantly reduced in *Opn4^−/−^* mice (two-way ANOVA, the star indicates light pulse×genotype interaction: *p*<0,001).

As expected from our previous work and that of others [16],[17], a 1-h light pulse administered during the dark period induced c-Fos immunoreactivity in the SCN in both genotypes (two-way-ANOVA, light-pulse effect: *p*<0.001, followed by post hoc Fisher protected least significant difference [PLSD]: *p*<0,05); however, the induction was half that observed in *Opn4^+/+^* mice (two-way ANOVA, light×genotype interaction: *p*<0,001; light-pulse effect: *Opn4^+/+^* vs. *Opn4^−/−^*, post hoc Fisher PLSD, *p*<0,05) (Figure 3). This reduction in c-Fos immunoreactivity has been functionally linked to the reduced ability to phase shift circadian rhythms in these mice. This does not, however, rule out the possibility that the reduced activation of SCN neurons could also contribute to the reduced direct effects of light on sleep and waking described here.

**Figure 4:**
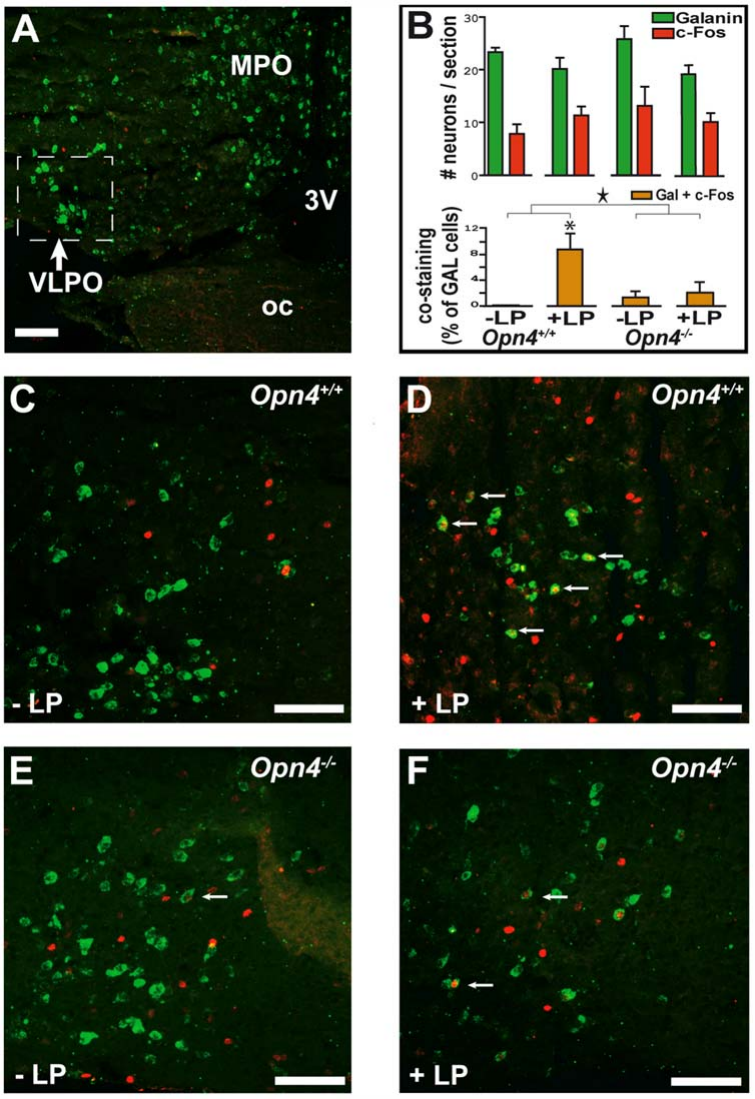
Effects of a 1-h light (L) pulse on c-Fos immunoreactivity in the VLPO. (A) “Sleep-active neurons” of the VLPO identified by ISH contain galanin mRNA (labeled green). (B) Top: histograms represent the number of VLPO neurons expressing galanin mRNA or c-FOS protein per section (mean±SEM). Bottom: VLPO costained (galanin+c-Fos) neurons expressed as a percentage of the total number of galanin mRNA-positive neurons (mean±SEM). A two-way ANOVA (with factors light pulse and genotype) revealed that the L pulse induced an increase in VLPO costained cells in *Opn4^+/+^*, but not in *Opn4^−/−^* mice (Two-way ANOVA, L-pulse effect: *p*<0,05; L pulse×genotype interaction: a star indicates *p*<0.05; the asterisk [*] indicates post hoc Fisher PLSD: *p*<0.05). The light-induced immunoreactivity in VLPO in wild-type animals is specific to galaninergic neurons and of a large magnitude; however, the proportion of c-Fos-stained galanin neurons is low (e.g., *Opn4^+/+^*: +L pulse: 9% of total number of galanin mRNA-containing cells). (C) In the absence of a L pulse (control condition at ZT16), c-Fos immunoreactivity was found in nongalanin mRNA-containing neurons in the VLPO of *Opn4^+/+^*. (D) The L-pulse-induced c-Fos expression (labeled red) in some of the galanin mRNA-positive (green) neurons of the VLPO in *Opn4^+/+^* (indicated by arrows). (E and F) In *Opn4^−/−^* mice, the same low number of galanin mRNA-positive neurons express c-Fos in both conditions, without (E) or with (F) light pulse. 3v, third ventricle; LP, L pulse administered during the habitual dark period (ZT15–16); MPO, medial preoptic area; oc, optic chiasm. Scale bars in (A) and (C–F) indicate 100 µm.

We further determined whether melanopsin conveys light information to the VLPO, in particular to the galaninergic sleep-active neurons, using double staining for c-FOS protein (immunohistochemistry) and for galanin mRNA (in situ hybridization). Whereas overall c-Fos immunoreactivity in the VLPO area was not significantly affected by the light pulse, in *Opn4^+/+^* mice, the percentage of galanin-containing (GAL) neurons coexpressing c-Fos did significantly increase compared to the control condition (i.e., darkness) (Figure 4). In contrast, in *Opn4^−/−^* mice, GAL c-Fos-costained neurons were present at low levels in both lighting schedules (2%), and no induction of c-Fos in GAL neurons was observed, suggesting that at least at this time of day, melanopsin-containing RGCs contribute significantly to the effects of light on the activity of these sleep-active VLPO neurons (two-way ANOVA, light-pulse effect: *p*<0.05, light×genotype interaction: *p*<0,05; light-pulse effect: *Opn4^+/+^*: *p*<0,05; *Opn4^−/−^*: *p* = 0,23, post hoc Fisher PLSD).

### Sleep and Quantitative ECoG Analyses under Standard Light–Dark Conditions

**Figure 5:**
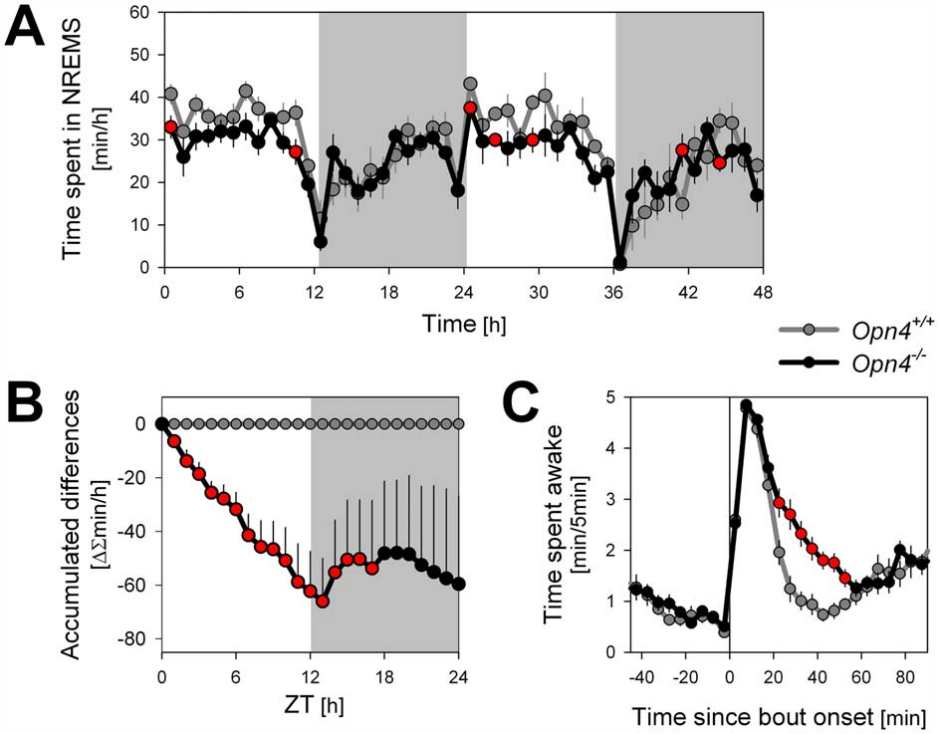
Time course of NREM sleep time and wake distribution under a standard LD 12∶12 schedule. (A) Despite a similar time course, NREM sleep levels attained during the 12-h light period were generally lower in *Opn4^−/−^* mice. (B) Dynamics of the accumulated differences demonstrate that *Opn4^−/−^* mice lose 1 h of NREM sleep per day, a loss incurred during the light period exclusively (average of the two baseline days; see also Table 1). (C) Sustained waking bouts in *Opn4^−/−^* are longer than in wild-type animals. See Table S1 for details on the definition of a sustained bout. Red-filled circles denote significant genotype differences (*p*<0.05; post hoc *t*-tests). Values represent mean±SEM (both days *n* = 9/genotype; for some mice the first or second day could not be included; *Opn4^+/+^*: day 1 *n* = 7, day 2 *n* = 5; *Opn4^−/−^* day 1 *n* = 9, day 2 *n* = 6).

The mouse is a nocturnal species that avoids light of higher intensity. *Opn4^−/−^* mice are no exception and, similar to their littermate wild-type controls (*Opn4^+/+^*), mostly sleep during the light period and are awake during the dark period of the LD 12∶12 cycle (Figure 5A; Table 1). Nevertheless, *Opn4^−/−^* mice lose ca. 1 h of NREM sleep per day relative to *Opn4^+/+^* mice. This marked loss of sleep occurred during the 12-h light period exclusively (Figure 5B) and resulted in an attenuation of the diurnal distribution of sleep and waking (Table 1). This genotype difference could be due to a reduced capacity of light to induce sleep or to suppress wakefulness, an interpretation underscored by the results of the light- and dark-pulse experiments (Figure 1A and 1B) and the LD 1∶1 experiment (Figure 2A). Indeed, for both genotypes, the levels of sleep reached during the 1-h light intervals during the subjective light period of the LD 1∶1 schedule were similar to those reached during the light period of the LD 12∶12 schedule; i.e., *Opn4^−/−^* mice slept less when light was present during the (subjective) light period of both the LD 1∶1 and LD 12∶12 (Table 1). Moreover, the sleep loss observed in *Opn4^−/−^* mice was associated with an overall deficit in ECoG delta power during NREM sleep (see below).

The wakefulness present during the light period is organized in more or less regularly occurring bouts lasting on average about 20–25 min [28] (Figure S1; Table S1). To examine in more detail the mechanism underlying the differences in sleep time during the light period, we analyzed these spontaneous waking bouts. In *Opn4^+/+^* mice, after 20 min of sustained wakefulness, waking values reverted quickly to the low values characteristic of the light period (Figure 5C). In contrast, in *Opn4^−/−^* mice, these waking bouts lasted on average 11 min longer (Table S1), and values remained above wild-type levels for 30 min (Figure 5C). The similarity between these results and the results observed after the single dark pulse (Figure 1B) suggest that the reduced capacity of light to suppress wakefulness and to induce sleep in *Opn4^−/−^* mice also contributed to an overall reduction in sleep time during the light period.

**Figure 6:**
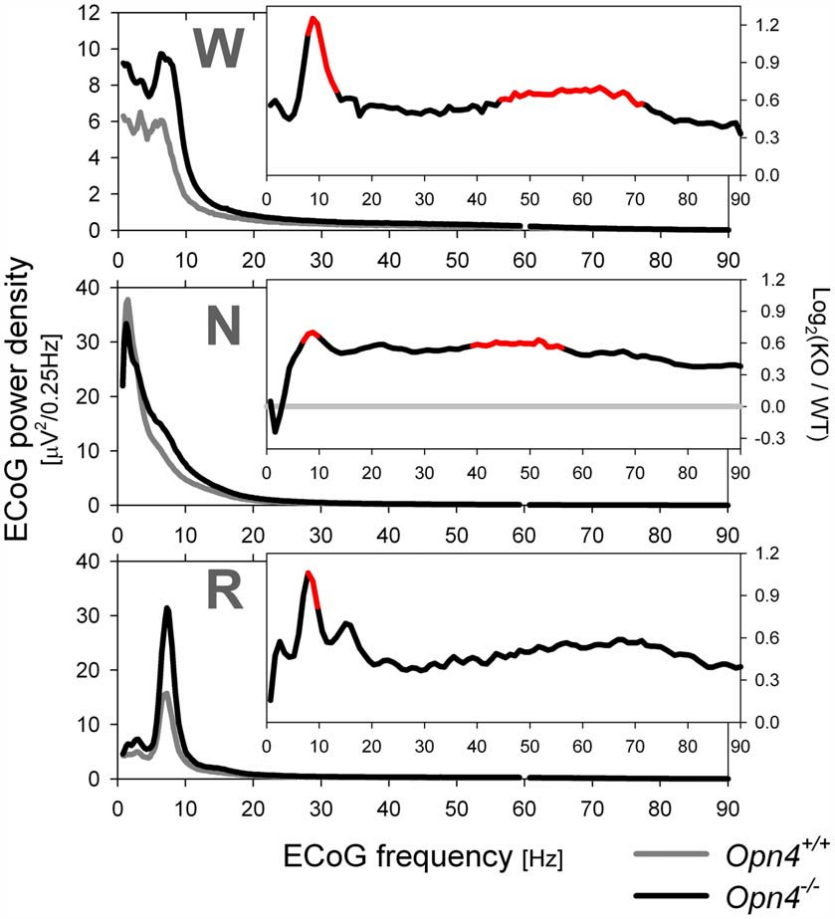
Average ECoG spectral profiles for each behavioral state recorded during 2 d under standard LD 12∶12 conditions. Power density in the theta and gamma frequency ranges was generally higher and in the delta frequencies lower (during NREM sleep) in *Opn4^−/−^* mice. Insets: relative spectral changes quantified as log_2_ of the ratio between *Opn4^−/−^* (KO) and *Opn4^+/+^* (WT) power density values (e.g., 1 signifies a two-fold increase). Line segments in red denote significant genotype differences (*p*<0.05; post hoc *t*-tests; *n* = 9/genotype). N, NREM sleep; R, REM sleep; W, wakfulness.

Quantitative analysis of the ECoG during the three behavioral states revealed an increase in ECoG power density in the theta and gamma frequency bands in *Opn4^−/−^* mice compared to in *Opn4^+/+^* mice (Figure 6). These differences were present both during the light and dark periods of LD 12∶12 (unpublished data). Such ECoG changes during NREM sleep are typically associated with reduced sleep quality [29], whereas during wakefulness, ECoG activity in these bands is associated with exploratory behavior, alertness, and cognition [25],[26],[30], and is thus markedly increased during waking compared to NREM sleep [31]. Genotype effects on theta activity could be analyzed in more detail in REM sleep because theta oscillations are especially prevalent and regular during this state [32]. Theta peak power during both REM sleep and wakefulness was nearly doubled in *Opn4^−/−^* mice compared to controls (Figure 6; Table S2). The frequency of the theta oscillation during REM sleep was faster in *Opn4^−/−^* mice compared to *Opn4^+/+^* mice during the light period, but not during the dark period. In mice, theta frequency during REM sleep is usually slower in the light compared to the dark period [32] (Table S2). In *Opn4^−/−^* mice, no evidence for such dark–light slowing was, however, found (Table S2), suggesting that the melanopsin pathway can directly or indirectly modulate hippocampal activity.

### Altered Homeostatic Regulation of Sleep in *Opn4^−/−^* Mice

**Figure 7:**
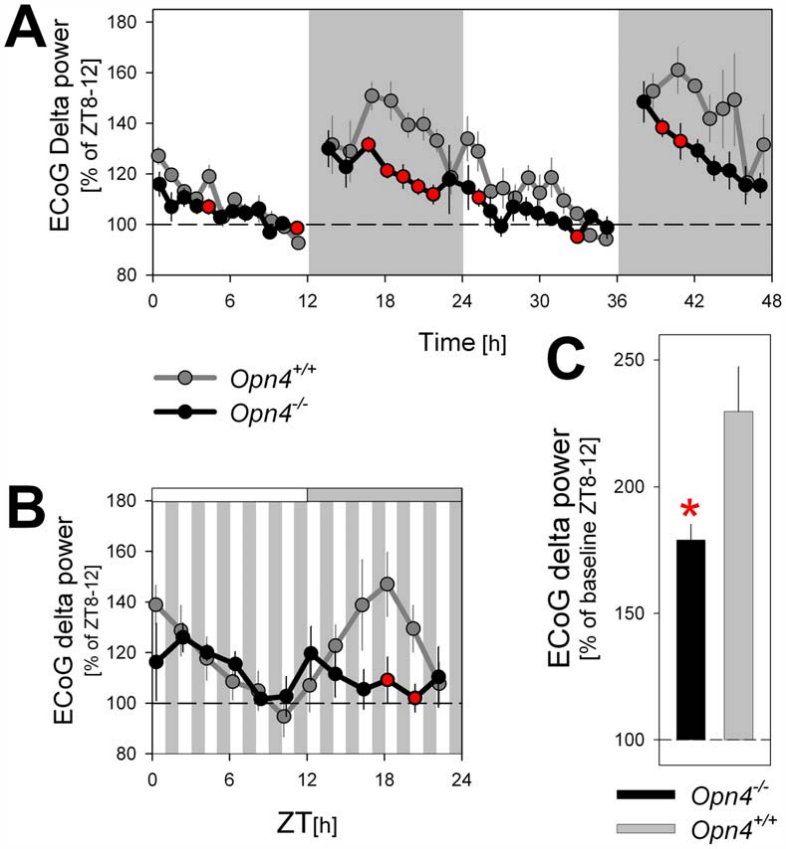
Time course of ECoG delta power (1–4 Hz) during NREM sleep under a standard LD 12∶12 cycle, a LD 1∶1 cycle, and during recovery sleep after sleep deprivation. (A and B) Time course analyses of ECoG delta power revealed that both under LD 12∶12 (A) and LD 1∶1 (B) schedules values were generally lower in *Opn4^−/−^* mice during the (subjective) dark periods. (C) Level of delta power reached after 6 h of sleep deprivation (starting at light onset; ZT0–6) was lower in *Opn4^−/−^* mice. Mean values (±SEM) were expressed as percentage of level reached during ZT8–12 of a preceding baseline day. Red filled circles and asterisk denote significant genotype differences (*p*<0.05; post hoc *t*-tests; (A) *n* = 9/genotype; (B) *n* = 4/3 for *Opn4^−/−^* and *Opn4^+/+^* mice, respectively; (C) *n* = 4/genotype).

Curtailing sleep time usually results in an increased need for sleep. It is therefore surprising that in *Opn4^−/−^* mice, levels of ECoG delta power during NREM sleep, a reliable correlate of sleep need, were generally lower while less time was spent in NREM sleep compared to *Opn4^+/+^* mice. This reduction in delta power was especially pronounced in the dark periods of the LD 12∶12 schedule (Figure 7A). The same difference was observed during the subjective dark period under the LD 1∶1 schedule (Figure 7B) and thus did not depend on the presence or absence of light. Moreover, this reduction was not associated with an increased fragmentation of sleep (Figure S3) that could have interfered with the expression of delta oscillations.

The unexpected reduction of delta oscillations observed in melanopsin-deficient mice might result from alterations in the properties of the sleep homeostat. We tested the possibility that the build-up of a pressure for sleep when animals are awake occurs at a slower rate in a sleep deprivation experiment. Mice of both genotypes were kept awake by gentle handling for 6 h starting at light onset (ZT0–6). The level of delta power reached during NREM sleep immediately following the sleep deprivation was significantly lower in *Opn4^−/−^* mice as compared to wild-type animals (Figure 7C), suggesting that in the absence of melanopsin, the dynamics of the sleep homeostat are altered.

## Discussion

Melanopsin-containing RGCs modulate a broad range of physiological responses to light, ranging from pupil constriction to circadian phase shifting. Here, we demonstrate that melanopsin-containing RGCs can also contribute to the acute light induction of sleep. Previously, it was assumed that only the rod–cone system was involved in these direct effects of light through conscious alerting. In humans, a role for melanopsin in the direct effects of light has already been suggested [33],[34] because the sensitivity of sleep–wake to acute light exposure is at a maximum at short wavelengths (460–480 nm), corresponding to melanopsin's peak light sensitivity at 480 nm. The application of blue light in normal subjects has been reported to activate multiple brain areas within seconds [35], and the observed responses to 1-h light or dark pulses quantified here in mice occur quickly as well (within minutes).

### Circadian Gating of the Direct Effects of Light

A main finding of our study is the result of the LD 1∶1 experiment that revealed that time of day modulated the acute effects of light and dark only in *Opn4^−/−^* mice. During a 7-h period of the subjective dark period, *Opn4^−/−^* mice did not respond to the ongoing LD alterations with respect to sleep–wake induction. During this time, we and others [20],[21] applied the single 1-h light pulse that has led to the conclusion that melanopsin mediates the direct effects of light. Our data show that at other times of day, melanopsin is not necessary for mediating these acute light effects on sleep duration.

Because light perception in *Opn4^−/−^* mice depends solely on rod–cone photoreception, it can be argued that the inability to respond to light at specific times of day results from circadian changes in rod–cone sensitivity. In intact mice, no time-of-day effect was observed in the response to the LD 1∶1 schedule, suggesting that melanopsin compensates for circadian changes in rod–cone sensitivity. Such interaction between the two photosensitive systems could take place in melanopsin-containing RCGs since these cells integrate rod–cone input important for mediating nonvisual light information to the brain [10]. Circadian modulation of retinal output has been described, and for example, variations in rod–cone electrical coupling were shown to lead to higher rod–cone photosensitivity during the dark period [36]. In contrast, another study showed higher cone sensitivity during the (subjective) light period, a circadian difference that required the presence of melanopsin [37]. It is unknown whether such effects exist also for the nonvisual, acute effects of light on sleep. Melanopsin protein levels are also known to vary across the day due to both circadian- and light-dependent influences reaching their highest levels during the light period and lowest during the dark period [38]. Assuming that differences in protein levels translate into differences in (blue) light sensitivity further complicates the issue and calls for further investigation.

We want to emphasize here that the above-described effects concern the acute effects of light observed at the transitions between lighting conditions. We presented evidence that melanopsin also affects several aspects of sleep during the sustained 12-h light period of the LD 12∶12 schedule, including the spectral composition of the ECoG, the frequency of theta oscillations during REM sleep, and waking bout duration. The genotype differences in the ECoG activation during the 1-h dark pulse was also observed during this period of the day.

### Neuronal Pathways Relaying the Direct Effects of Light on Sleep

Although circadian gating already occurs at the level of the retina [36],[39],[40], the 50% reduction in the activation of SCN neuronal activity after a light pulse in *Opn4^−/−^* mice may also be relevant for the circadian variation of the direct effects of light in these mice. As SCN functionality is conserved in the absence of melanopsin [9], a potential role of the SCN in also mediating the acute, noncircadian effects of light could explain the time-of-day-dependent variation in light sensitivity. The SCN is well placed to relay such activation since it influences key areas for sleep–wake control, such as the VLPO.

Besides the SCN, other anatomical targets of melanopsin-containing RGCs have been identified [22],[24],[41]. Among the various projection areas, the VLPO is a good candidate for relaying light information to sleep–wake neuronal networks. The VLPO has been implicated in the induction of NREM sleep [42], and galanin- and GABA-releasing neurons within the VLPO have been functionally characterized as a cluster of sleep-active neurons. These sleep-active neurons are thought to actively promote sleep by inhibiting the ascending arousal systems [43],[44]. A regional analysis of *c-Fos* expression within the VLPO area was conducted by Lupi et al., 2008, by real time PCR from punched tissues [21]. This analysis revealed a 2-fold increase in *c-Fos* following a 1-h light pulse in wild-type mice that was absent in *Opn4^−/−^* mice. Using a different method (i.e., counting the number of c-FOS immunoreactive cells in the VLPO), we found little evidence for a light-induced general activation of the VLPO area. Only the costaining of galanin and c-Fos used in our study allows for an evaluation of transcriptional changes specific to those VLPO neurons that could mediate the sleep-promoting effects of light. Here, we provide the first evidence that light indeed activates galanin-containing cells in the VLPO. This light-induced c-Fos immunoreactivity was absent in *Opn4^−/−^* mice. This anatomical result is consistent with the complete absence of sleep–wake changes during the 1-h light pulse at this time of day, suggesting that the VLPO might play a role in mediating these effects of light on sleep. Our findings thus suggest that light directly impinges on VLPO sleep-active neurons thereby shifting the balance of the reciprocal inhibitory interaction towards sleep promotion and arousal inhibition.

### Melanopsin Modulates the Induction of the ECoG Correlates of Alertness and Cognition

The rapid induction of ECoG activity in both the theta and gamma frequency bands during the dark pulse in *Opn^+/+^* mice is consistent with the alerting effects of light-to-dark transitions in a nocturnal species. Theta oscillations accompany exploratory behavior [45] and are also involved in long-term potentiation (LTP) and learning [46]–[48]. Gamma activity accompanies a wide variety of cognitive processes, including perceptual processing, attention, arousal, object recognition, and in humans, language perception [49]. As in the present study, gamma activity is often associated with the presence of theta oscillation in rodents [50]. The downstream events initiated by melanopsin have been suggested to affect LTP as well as performance in learning and memory tasks [51]. In humans, nonvisual responses related to alertness and cognition are associated with changes in regional brain activity detected by positron emission tomography (PET) or functional magnetic resonance imaging (fMRI) [52],[53]. Application of blue light during a working memory task induces specific brain activity changes within a time frame of seconds [7].

### A Role for Melanopsin in Sleep Homeostasis?

ECoG delta power is widely used to reflect a sleep homeostatic process because its level monotonically increases with wake duration and decreases during NREM sleep [54]. The reduction of ECoG delta power in *Opn4^−/−^* mice was pronounced and present during both the LD 12∶12 and LD 1∶1 schedules. This reduction was all the more surprising because NREM sleep time was reduced in these mice, and reduced sleep time is usually associated with increased delta power. The unexpected reduction of delta oscillations in melanopsin-deficient mice might result from alterations in the properties of the sleep homeostat. One possibility is that NREM sleep may have been more efficient in reducing sleep need in *Opn4^−/−^* mice. However, by comparing the ECoG spectral profiles during NREMS, evidence to the contrary was found; the increase in theta and gamma activity combined with reduction of delta activity in *Opn4^−/−^* mice indicate that NREM sleep seems less profound and, if anything, likely to be less efficient in reducing sleep need and ECoG delta power [55]. The other possibility is that the buildup of a pressure for sleep when animals are awake occurs at a slower rate. We confirmed this possibility in a sleep-deprivation experiment; the level of delta power reached during NREM sleep immediately following the sleep deprivation was significantly lower in *Opn4^−/−^* mice as compared to wild-type animals. In fact, delta power levels reached after sleep deprivation in *Opn4^−/−^* mice were as low as the lowest levels observed in a panel of six inbred strains of mice [56]. This reduced compensatory response to sleep loss suggests that indeed the need for sleep increases at a slower rate in *Opn4^−/−^* mice. The experiment also demonstrates that the reduced delta power is not specific to lighting condition or time of day.

Although a role for melanopsin in sleep homeostasis is unexpected and not easily reconciled with current hypotheses on sleep homeostasis, the modulation of VLPO sleep-active neurons by melanopsin-containing RGCs could hint to a possible mechanism. Sleep-active neurons in the VLPO are thought to be a neuronal substrate of sleep homeostasis because more than 50% of galanin-containing neurons express c-Fos during recovery sleep after a sleep deprivation [57]. A final consideration that applies to all studies using noninducible loss-of-function mutants is that the altered relationship between the sleep–wake distribution and ECoG delta power reflects a developmental effect.

### Conclusions

Our results provide evidence that melanopsin-containing RGCs contribute to the noncircadian, nonvisual direct effects of light on sleep and the ECoG correlates of alertness and cognition. Melanopsin's contribution to the acute affects of light on sleep duration was however limited to a ca. 7-h time window. Our findings suggest that the acute photic sleep promotion stems, at least partly, from a stimulation of VLPO sleep-active neurons, which in return, would lead to an inhibition of the arousal systems. Apart from these direct effects of light observed at the transitions between lighting conditions, the daily loss of NREM sleep over the 12-h light period in *Opn4^−/−^* mice provides evidence that melanopsin also modulates the expression of sleep under sustained periods of light exposure. If confirmed in humans, our observations concerning the time-dependent effects of melanopsin's contribution to the acute effects of (blue) light as well as the effects of sustained light exposure will have applications for the clinical use of light therapy as well as for 24-h patterns in luminance [58]. Finally, the discovery that the homeostatic regulation of sleep need can be affected by a photopigment is intriguing and represents a novel concept in the field of sleep regulation.

## Materials and Methods

### Animals

All experiments were performed on adult male *Opn4^−/−^* mice and wild-type littermates (as controls), and carried out in accordance with the National Institutes of Health Guide for the Care and Use of Laboratory Animals as well as local veterinary office and use committees at Stanford University (for details and genotyping, see Text S1 and Ruby et al. (2002) [17]).

### ECoG Recordings and Analyses

The methods concerning the recording and analysis of the ECoG in mice are described in detail in Text S1 and elsewhere [32],[59]. Briefly, ECoG and electromyogram (EMG) recordings from mice implanted with a classical set of electrodes, were collected (using commercial hardware, EMBLA, and software, Somnologica-3) for the following conditions: (1) two continuous baseline days under LD 12∶12 cycle; (2) a 1-h light pulse (white fluorescent tubes, Philips F32T8/TL741 Hi-Vision 40 W, 4,100 K [Philips Lighting], broad spectrum, mainly 400–800 nm) administered 3 h after dark onset (ZT 15), (3) 1-h dark pulse administered 3 h after light onset (ZT 3), (4) a 1∶1 LD cycle for 24 h, and (5) 6-h sleep deprivation starting at light onset. The behavior in each 4-s epoch was classified as waking, REM sleep, or NREM sleep based on the ECoG and EMG signals according to standard criteria [32]. The ECoG signal (analog-to-digital converted) was subjected to discrete Fourier transform (DFT) yielding power spectra at 0.25-Hz resolution. For each state, an ECoG spectral profile (0 to 90 Hz) was constructed by averaging all 4-s epochs scored as that state.

### In Situ Hybridization (ISH) and Immunohistochemistry (IHC)

Galanin ISH and c-Fos immunostaining on frozen sections with or without (control condition) a prior 1-h light exposure was carried out as described previously [60],[61] and are detailed in Text S1. Staining of GAL mRNA was chosen instead of staining the protein because it has been shown in mice that cell body staining of galanin-containing neurons by IHC is only reliable in colchicine-treated animals. In *Opn4^+/+^* and *Opn4^−/−^* mice (*n* = 6 of each genotype), identification of c-Fos immunoreactive cells and sacrifice of animals occurred at the conclusion of a 1-h light pulse as well as without a light pulse as a negative control. Sleep was recorded in these animals, several weeks before, under a light pulse identical to the light pulse administered the day of the perfusion to confirm that their response to light was similar to those of the whole group. Before ISH series of 18-µm-thick sections were pretreated by an antigen retrieval procedure [62]. The mouse galanin probe (National Center for Biotechnology Information [NCBI] BC044055, covering a 716-base sequence of the GAL prepro-mRNA) was used in a dilution of 1∶500. The ISH protocol preceding the immunohistochemical protocol was identical to the procedure described previously [60],[61]. GAL mRNA was visualized using a horse radish peroxidase (POD)-labeled sheep-anti-digoxiginin antibody (Roche 1207733, diluted 1∶200), and Alexa-tyramide 488 (Molecular Probe; diluted 1∶100). Hereafter, sections were incubated with a rabbit anti-c-Fos antiserum (c-Fos antibody dilution: 1∶500; code no: 9412, [23] and visualized by Alexa568-conjugated goat anti-rabbit antibody (Molecular Probe; diluted 1∶1,000). Counting of GAL- and c-Fos-expressing neurons in the VLPO and SCN was conducted using a confocal microscope (Zeiss LSM 510; Brock and Michelsen) equipped with appropriate filter settings for detecting Alexa488 and Alexa568 was used. The quantification method is detailed in Text S1.

### Statistical Analysis

Differences in sleep amounts and quantitative EEG variables were determined by single- or multiple-way ANOVAs, followed by post hoc *t*-tests if 5% significance levels were reached. The differences in number of c-Fos, Gal; or c-Fos+Gal neurons were assessed by two-way (light and genotype conditions) ANOVA, followed by post hoc Fisher PLSD.

## Supporting Information

Figure S1 **Overview of wakefulness expressed per 5-min intervals in one** ***Opn4^−/−^*** **(left) and one wild-type (right) animal.** Shown are the various LD regimens used, including two consecutive days of baseline (top), 1-h dark pulse administered at ZT3 and 1-h light pulse administered at ZT15 (middle) and a 24-h d under a 1-h∶1-h LD cycle (bottom). A minimum of 10 d was allowed between each experimental condition. Recordings started 1 d prior to each condition to verify that sleep–wake amounts and architecture returned to baseline values.
(1.08 MB TIF)

Figure S2 **Heat map of the light (L) and dark (D) and time-of-day-dependent changes in time spent awake under the LD 1∶1 schedule (see** **Figure 2B** **).** Waking values (waking minutes/5-min intervals; warmer colors correspond to more waking/5 min) over 3 h were aligned according to the onset (0 h; grey horizontal bars) of the 1-h dark periods. Only in *Opn4^−/−^* mice does the capacity of the light and dark pulses to shape the sleep–wake distribution vary with time of day. This is especially clear between ZT15 and ZT21 during the subjective dark period (ZT12–24; grey vertical bars). Note that values depicted between time 1 and 2 at one ZT corresponds to the values between −1 and 0 of the subsequent ZT interval. Also note that *Opn4^+/+^* mice learn to anticipate dark-period onset as the day progresses.
(4.02 MB TIF)

Figure S3 **Relative frequency distribution of episode duration of NREM sleep (N), REM sleep (R), and waking (W) episodes under standard LD 12∶12 conditions.** Vertical bars represent the number of episodes (mean±standard error of the mean [SEM]) expressed per hour of time spent in each state for nine categories of episode duration. For none of the behavioral states did genotype affect the distribution.
(0.55 MB TIF)

Table S1 **Duration and number of sustained waking bouts during baseline recordings under a LD 12∶12 schedule.** Sustained waking bouts in *Opn4^−/−^* are longer than in wild-type animals (*Opn4^+/+^*) during the 12-h light period (*p*<0.05; post hoc *t*-tests). See Text S1 below for selection criteria of sustained waking bouts. All values represent means±1 SEM (both days *n* = 9/genotype; for some mice the first or second day could not be included; *Opn4^+/+^*: day 1 *n* = 7, day 2 *n* = 5; *Opn4^−/−^* day 1 *n* = 9, day 2 *n* = 6).
(0.03 MB DOC)

Table S2 **ECoG theta activity during REMS differed between genotypes.** ECoG power density at peak frequency was higher both in absolute and relative terms (not shown, but see Figure 6) in *Opn4^−/−^* mice. In the light period, theta oscillated at a higher frequency in *Opn4^−/−^* mice, reaching values normally attained during the dark period. As a result, the normal LD difference in theta peak frequency was absent in *Opn4^−/−^* mice. An asterisk (*) indicates significant genotype differences; a section mark (§) indicates significant LD differences (*p*<0.03; post hoc *t*-test). Values represent mean±SEM (light period: *n* = 8 and 9; dark period: *n* = 7 and 7, for *Opn4^−/−^* and *Opn4^+/+^*, respectively).
(0.03 MB DOC)

Text S1 **Supplemental experimental procedures: detailed procedures.**
(0.04 MB DOC)

## Abbreviations

ECoG: electrocorticogram
EEG: electroencephalogram
LD: light–dark
NREM: non-rapid eye movement
PLSD: protected least significant difference
REM: rapid eye movement
RGC: retinal ganglion cell
SCN: suprachiasmatic nucleus
VLPO: ventrolateral preoptic
ZT: Zeitgeber time
