# Supplementary figures and images for: Melanopsin as a Sleep Modulator: Circadian Gating of the Direct Effects of Light on Sleep and Altered Sleep Homeostasis in Opn4−/− Mice
Source: PLoS Biol. 2009 Jun 9;7(6):e1000125. doi: 10.1371/journal.pbio.1000125 (PMC2688840; doi:10.1371/journal.pbio.1000125)

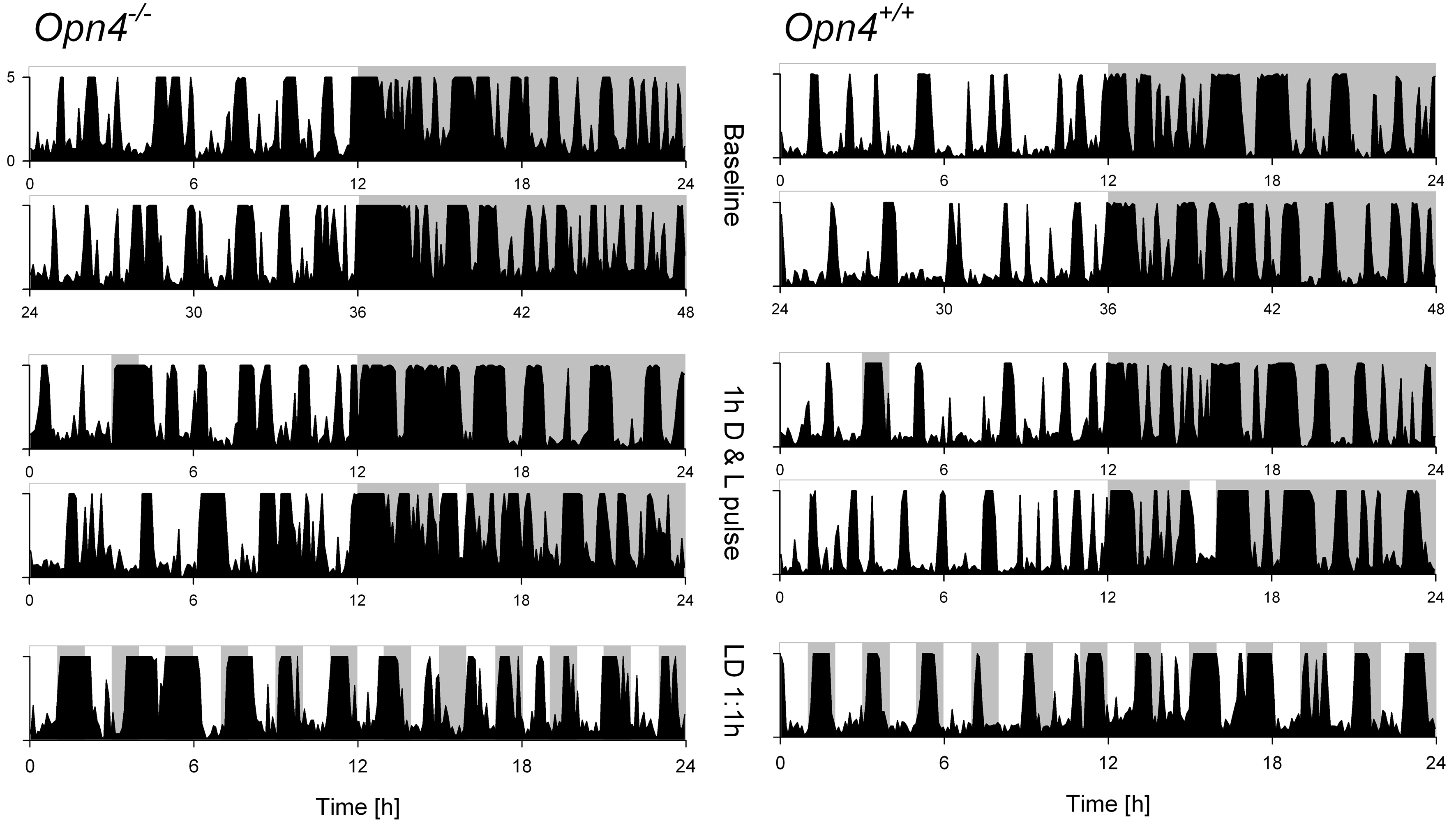

Supplement: Figure S1 — Overview of wakefulness expressed per 5-min intervals in one Opn4−/− (left) and one wild-type (right) animal. Shown are the various LD regimens used, including two consecutive days of baseline (top), 1-h dark pulse administered at ZT3 and 1-h light pulse administered at ZT15 (middle) and a 24-h d under a 1-h∶1-h LD cycle (bottom). A minimum of 10 d was allowed between each experimental condition. Recordings started 1 d prior to each condition to verify that sleep–wake amounts and architecture returned to baseline values. (1.08 MB TIF) [file pbio.1000125.s001.tif]

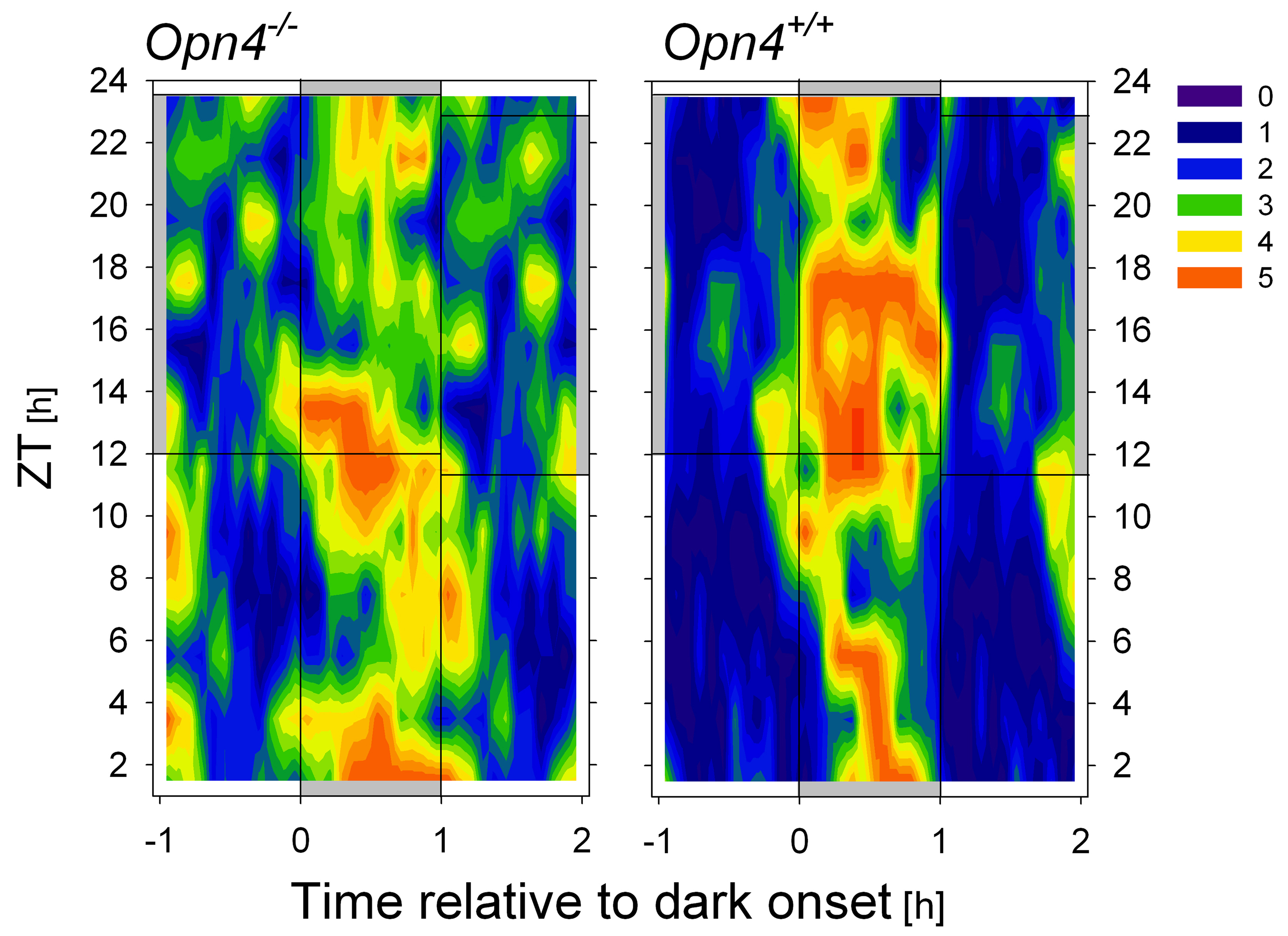

Supplement: Figure S2 — Heat map of the light (L) and dark (D) and time-of-day-dependent changes in time spent awake under the LD 1∶1 schedule (see Figure 2B ). Waking values (waking minutes/5-min intervals; warmer colors correspond to more waking/5 min) over 3 h were aligned according to the onset (0 h; grey horizontal bars) of the 1-h dark periods. Only in Opn4−/− mice does the capacity of the light and dark pulses to shape the sleep–wake distribution vary with time of day. This is especially clear between ZT15 and ZT21 during the subjective dark period (ZT12–24; grey vertical bars). Note that values depicted between time 1 and 2 at one ZT corresponds to the values between −1 and 0 of the subsequent ZT interval. Also note that Opn4+/+ mice learn to anticipate dark-period onset as the day progresses. (4.02 MB TIF) [file pbio.1000125.s002.tif]
