# Supplementary material for: Melanopsin as a Sleep Modulator: Circadian Gating of the Direct Effects of Light on Sleep and Altered Sleep Homeostasis in Opn4−/− Mice
Source: PLoS Biol. 2009 Jun 9;7(6):e1000125. doi: 10.1371/journal.pbio.1000125 (PMC2688840; doi:10.1371/journal.pbio.1000125)
Supplement: Table S1 — Duration and number of sustained waking bouts during baseline recordings under a LD 12∶12 schedule. Sustained waking bouts in Opn4−/− are longer than in wild-type animals (Opn4+/+) during the 12-h light period (p<0.05; post hoc t-tests). See Text S1 below for selection criteria of sustained waking bouts. All values represent means±1 SEM (both days n = 9/genotype; for some mice the first or second day could not be included; Opn4+/+: day 1 n = 7, day 2 n = 5; Opn4−/− day 1 n = 9, day 2 n = 6). (0.03 MB DOC) [file pbio.1000125.s004.doc]

|  | 12h L-period | | 12h D-period | |
| --- | --- | --- | --- | --- |
|  | number | length [min] | number | length [min] |
| *Opn4^-/-^* | 6.7 ± 0.3 | 34.3 ± 2.2 | 6.2 ± 0.4 | 66.6 ± 4.6 |
| *Opn4^+/+^* | 7.2 ± 0.5 | 23.3 ± 1.0 | 6.0 ± 0.5 | 77.9 ± 9.4 |
| *P* | *0.42* | *0.0003* | *0.73* | *0.30* |
