# Supplementary material for: Melanopsin as a Sleep Modulator: Circadian Gating of the Direct Effects of Light on Sleep and Altered Sleep Homeostasis in Opn4−/− Mice
Source: PLoS Biol. 2009 Jun 9;7(6):e1000125. doi: 10.1371/journal.pbio.1000125 (PMC2688840; doi:10.1371/journal.pbio.1000125)
Supplement: Table S2 — ECoG theta activity during REMS differed between genotypes. ECoG power density at peak frequency was higher both in absolute and relative terms (not shown, but see Figure 6) in Opn4−/− mice. In the light period, theta oscillated at a higher frequency in Opn4−/− mice, reaching values normally attained during the dark period. As a result, the normal LD difference in theta peak frequency was absent in Opn4−/− mice. An asterisk (*) indicates significant genotype differences; a section mark (§) indicates significant LD differences (p<0.03; post hoc t-test). Values represent mean±SEM (light period: n = 8 and 9; dark period: n = 7 and 7, for Opn4−/− and Opn4+/+, respectively). (0.03 MB DOC) [file pbio.1000125.s005.doc]

|  | *Opn4*  genotype | 12h Light | 12h Dark |
| --- | --- | --- | --- |
| Peak frequency  [Hz] | *–/–* | 7.28 ± 0.07* | 7.30 ± 0.10 |
|  | *+/+* | 6.93 ± 0.10 | 7.34 ± 0.07§ |
| Peak power  [μV^2^/0.25] | *–/–* | 31.9 ± 4.5* | 36.3 ± 4.5* |
|  | *+/+* | 14.6 ± 3.2 | 13.7 ± 4.3 |
