# Supplementary material for: Melanopsin as a Sleep Modulator: Circadian Gating of the Direct Effects of Light on Sleep and Altered Sleep Homeostasis in Opn4−/− Mice
Source: PLoS Biol. 2009 Jun 9;7(6):e1000125. doi: 10.1371/journal.pbio.1000125 (PMC2688840; doi:10.1371/journal.pbio.1000125)
Supplement: Text S1 — Supplemental experimental procedures: detailed procedures. (0.04 MB DOC) [file pbio.1000125.s006.doc]

# Supplemental Experimental Procedures: Detailed Procedures

# Animals:

The animals, originally obtained from Deltagen Laboratory, were reared under control environmental conditions (12h:12h light-dark; 25 ± 0.3° C, food and water ad libitum) and handled in agreement with the ethical rules for experimentation on laboratory animals in accordance with the National Institutes of Health Guide for the Care and Use of Laboratory Animals. The experimental protocols were approved by the local veterinary office and use committees at Stanford University. Genotype was validated by PCR (primers: Mel4: 5’–GCT CAC TAT ACC CTA GGC AC–3’; Mel2: 5’–GTC CAT GGC TAT GGC TGT CA–3’; TodoNeo1: 5’–CCG CTT TTC TGG ATT CAT CGA C–3’from Integrated DNA Technologies, Inc.) as described previously [1].

**ECoG Recordings and Analyses:**

Surgical preparation occurred under anesthesia delivered intraperitoneally with Nembutal (68 mg/kg; Stanford University IRB-approved). Adult male *Opn4^–/–^* (n=10) and *Opn4^+/+^* (n=10) mice were implanted with two ECoG and two EMG electrodes for sleep recordings at 10-12 weeks of age. Mice were allowed at least 14 days of recovery from surgery and habituation to experimental conditions before the experiments. Two undisturbed baseline days (48h) were recorded using commercial hardware (EMBLA™) and software (Somnologica-3™) under LD12:12h conditions (white fluorescent lights, 100lux, measured at the bottom of the cage). Continuous sleep recordings were also performed under the following conditions: (i) a 1 hour white-light pulse of (200 lux;) administered 3 hours after dark onset (ZT 15), (ii) 1-hour dark pulse administered 3 hours after light onset (ZT 3), and (iii) a 1-1 LD cycle for 24 hours. In a subset of mice a 6h sleep deprivation was performed at the end of all experiments. Sleep deprivation was performed by gentle handling [2] starting at light onset (ZT0-6). These experiments took place on separate days with at least 14 days in 12-12 LD between each experiment. The day of recording preceding the experimental condition was used to confirm that sleep-wake amounts and architecture had returned to baseline conditions. Both genotypes were recorded at the same time.

ECoG and EMG signals were amplified, filtered, and analog-to-digital converted at 2000Hz, subsequently down-sampled and stored at 200Hz. The ECoG signal was subjected to Discrete-Fourier Transform (DFT) yielding power spectra between 0 and 90 Hz (0.25Hz resolution) using a 4-s window. The behavior in each of these 4-s epochs was classified as waking, rapid-eye-movement (REM) sleep, or non-REM (NREM) sleep by visual inspection of the EEG and EMG signals without knowledge of the recording and genotype condition according to standard criteria [3]. Four-second epochs containing ECoG artifacts were marked so they could be excluded from ECoG spectral analyses. Genotype differences in sleep amount were calculated by averaging time spent in each state over 5min, and 1-, 12-, and 24h intervals. The frequency distribution of episode duration of NREMS, REMS, and waking was calculated as described previously [2]. In addition, the sustained waking bouts were defined by using a moving average (10-min window, 4-s increments) according to Franken et al. (2006) [4]. Ten-minute windows in which waking prevailed (>75%) were deemed "awake".  Awake windows separated by <20 min were merged into a single waking bout.

To determine the amplitude of the light-dark dependent changes in wakefulness under the LD1:1 schedule, sine-waves were fitted to the individual 5min values for wakefulness obtained in the 1h before, during, and after the 1h dark periods. Within subjects, the 5min values for the 6 dark-pulses given during the 12h subjective light period and the 6 dark-pulses given during the 12h subjective dark period were averaged. Sine-waves were fitted to the average time courses using SAS (SAS Institute Inc, Cary, NC; Proc NLIN) with amplitude, phase, y-offset as free parameters and period set to 2 hours (see Fig.2B). Thus obtained individual amplitudes for the subjective light and dark period were further analyzed using a 2-way ANOVA with factor genotype and repeated measures for factor light or dark period.

For each state an ECoG spectral profile was constructed by averaging all 4-s epochs scored as that state. The frequency range 59-61 Hz was omitted due to power-line artifacts in some of the recordings. Time dependent changes in ECoG power in specific frequency bands was performed for delta (1-4Hz) in NREM sleep, and theta (6-10Hz) and gamma (40-70Hz) during wakefulness. ECoG delta power during NREMS was normalized by expressing all values relative to the mean value obtained in the last 4h of the (subjective) light period. Determination of theta peak frequency and theta peak power in the REMS ECoG was performed according to Franken et al. (1998)[3].

**In Situ Hybridization (ISH) and Immunohistochemistry (IHC)**

In *Opn4^+/+^* and *Opn4^–/–^* mice (n=6/genotype), identification of c-Fos immunoreactive cells and sacrifice of animals occurred at the conclusion of a 1h light pulse and without light pulse as a negative control. Sleep was recorded in these animals, several weeks before, under a light pulse identical to the light pulse administered the day of the perfusion to confirm that their response to light was similar to those of the whole group. At the end of the 1h light pulse (ZT16), mice were deeply anesthetized with CO_2_ and perfused with heparin/NaCl followed by transcardial fixation for 15 min with 4% paraformaldehyde in PBS, pH 7.4 for in situ hybridization and combined immunohistochemistry. After dehydration in 30% sucrose for 48h the brains were frozen and cut in a freezing microtom as 18µm thick sections in series of four containing the VLPO and the SCN, respectively.

Before ISH all sections were pretreated by an antigen retrieval procedure as described by the manufacturer (DAKO TechMate 500/1000, Copenhagen, Denmark) using antigen retrieval buffer (DAKO ChemMate, code No. S 203120) in distilled water. The mouse galanin (GAL) probe used was obtained from NCBI (BC044055, cDNA clone MGC:54666, base 1 to 716 of the GAL prepro-mRNA inserted into a pCMV-SPORT6). Antisense and sense probes were labelled with digoxiginin using T7 and SP6 polymerase and used in a dilution of 1:500. The ISH protocol preceding the immunohistochemical protocol were identical to the procedure described previously [5,6]. GAL mRNA was visualized using a horse radish peroxidase (POD)-labelled sheep-anti-digoxiginin antibody (Roche 1207733, diluted 1:200), and Alexa-tyramide 488 (Molecular Probe diluted 1:100). Hereafter sections were washed and incubated over night with a well-characterized rabbit anti-c-FOS antiserum (c-Fos antibody dilution: 1:500; (code #9412) raised against amino acids 4-17 of the human/rat proteins [7]; kindly donated by Dr. Philip J. Larsen, The Panum Institute, Copenhagen, Denmark; see [8] and visualized by Alexa568 conjugated goat anti-rabbit antibody (Molecular Probe, diluted 1:1000). Hybridization with the GAL sense probe gave no specific labeling (data not shown).

To investigate the number of GAL expressing neurons in the VLPO also expressing c-Fos immunoreactivity a confocal microscope (Zeiss LSM 510, Brock and Michelsen, Birkerød, Denmark) equipped with appropriate filter settings for detecting Alexa488 and Alexa568 was used. The number of GAL expressing neurons in the VLPO cluster was counted by an experimenter blind to experimental conditions, in an area of 225µm x 225µm at a level corresponding to bregma -0.1mm [9] (Figure 2 ). Two brain sections from each animal representing the VLPO area were analyzed and cells containing GAL, c-Fos and GAL+c-Fos was manually determine using a grid on each image.

Sections from each animal containing the SCN were used as control of the light stimulation paradigm. SCN was stained for arginin-vasopressin (AVP antibody was raised in guinea pig (code no.: GHC 8103, Peninsula Laboratories, San Carlos, CA, USA, dilutes 1:1000) and co-stained for c-Fos which is strongly induced in the SCN after light stimulation at night [10]. C-Fos was visualized as described above and AVP as described previously [11]. The number of c-Fos immunoreactive neurons in the SCN was counted the same way as above. Elimination of the primary antisera eliminates all specific staining.

**Supplemental References:**

1. Ruby NF, Brennan TJ, Xie X, Cao V, Franken P, et al. (2002) Role of melanopsin in circadian responses to light. Science 298: 2211-2213.

2. Franken P, Malafosse A, Tafti M (1999) Genetic determinants of sleep regulation in inbred mice. Sleep 22: 155-169.

3. Franken P, Malafosse A, Tafti M (1998) Genetic variation in EEG activity during sleep in inbred mice. Am J Physiol 275: R1127-1137.

4. Franken P, Dudley CA, Estill SJ, Barakat M, Thomason R, et al. (2006) NPAS2 as a transcriptional regulator of non-rapid eye movement sleep: genotype and sex interactions. Proc Natl Acad Sci U S A 103: 7118-7123.

5. Bourgin P, Huitron-Resendiz S, Spier AD, Fabre V, Morte B, et al. (2000) Hypocretin-1 modulates rapid eye movement sleep through activation of locus coeruleus neurons. J Neurosci 20: 7760-7765.

6. Hannibal J, Fahrenkrug J (2002) Melanopsin: a novel photopigment involved in the photoentrainment of the brain's biological clock? Ann Med 34: 401-407.

7. Woldbye DP, Greisen MH, Bolwig TG, Larsen PJ, Mikkelsen JD (1996) Prolonged induction of c-fos in neuropeptide Y- and somatostatin-immunoreactive neurons of the rat dentate gyrus after electroconvulsive stimulation. Brain Res 720: 111-119.

8. Hannibal J, Fahrenkrug J (2004) Target areas innervated by PACAP-immunoreactive retinal ganglion cells. Cell Tissue Res 316: 99-113.

9. Franklin KaPG (1997) The mouse brain in stereotaxic coordinates: Academic Press.

10. Kornhauser JM, Nelson DE, Mayo KE, Takahashi JS (1990) Photic and circadian regulation of c-fos gene expression in the hamster suprachiasmatic nucleus. Neuron 5: 127-134.

11. Sanggaard KM, Hannibal J, Fahrenkrug J (2003) Serotonin inhibits glutamate- but not PACAP-induced per gene expression in the rat suprachiasmatic nucleus at night. Eur J Neurosci 17: 1245-1252.
